# Primary breast tuberculosis: imaging findings of a rare disease

**DOI:** 10.1186/s13244-021-00961-3

**Published:** 2021-02-15

**Authors:** Ali H. Baykan, Hakan S. Sayiner, Ibrahim Inan, Elcin Aydin, Sukru M. Erturk

**Affiliations:** 1 Department of Radiology, Faculty of Medicine, Adiyaman University, Yunus Emre Mahallesi 1164 Sokak No:13, 02200 Merkez/Adiyaman, Turkey; 2 Department of Infectious Diseases and Clinical Microbiology, Faculty of Medicine, Adiyaman University, Adiyaman, Turkey; 3 Department of Radiology, Biruni University Hospital, Istanbul, Turkey; 4 Zubeyde Hanim Hospital Izmir, Faculty of Medicine, Baskent University, Izmir, Turkey; 5 Department of Radiology, Faculty of Medicine, Istanbul University, Istanbul, Turkey

**Keywords:** Breast, Tuberculosis, Extrapulmonary tuberculosis, Mammography

## Abstract

Breast tuberculosis is a rare form of extrapulmonary tuberculosis which tends to affect females of reproductive age, and is much rarer in males, postmenopausal women, and pre-pubescent girls. It is difficult to diagnose because it can mimic benign conditions such as a fibroadenoma, as well as malignant diseases like a carcinoma. It is also particularly difficult to distinguish breast tuberculosis from granulomatous mastitis, so other possible diagnoses should be ruled out first. The diagnosis of breast tuberculosis has three essential pillars: clinical examination, radiological evaluations, and histopathological sampling. Radiological evaluations are not only critical in diagnosis but are also important in the planning of the treatment and during the follow-up. This paper aims to review the imaging findings and the differential diagnosis of breast tuberculosis.

## Key points

- Breast tuberculosis is a rare type of extrapulmonary tuberculosis.
- Breast tuberculosis is recognized, but an uncommon cause of a breast mass.
- Awareness of this condition is essential because it can clinically, and radiologic imaging mimic breast carcinoma.

## Introduction

Tuberculosis is an infectious disease caused by *Mycobacterium tuberculosis* bacillus, and which can survive in the macrophages of the host [1]. Tuberculosis is more common in developing countries, and its incidence increases among immunosuppressed individuals [2–6].

Although tuberculosis primarily attacks the lungs, other organs are also at risk of infection [7]. The prominent English surgeon Sir Astley Cooper reported the first case of tuberculous mastitis in 1829, describing it as a “scrofulous swelling of the bosom.” [8].

Breast tuberculosis accounts for less than 0.1% of all breast pathologies [2, 9] and has been reported as 3–4.5% of breast pathologies requiring surgery in developing countries [9, 10]. Multipara and lactating women between the ages of 20 and 40 are more frequently affected [4, 5, 11–13], while trauma and immunosuppressive conditions are among the other risk factors [3]. Breast tuberculosis can be either primary or secondary, but primary breast tuberculosis is extremely rare. If there is no other focus of infection elsewhere in the body, it is considered primary [13, 14].

## Background

Breast parenchyma is resistant to the tuberculosis bacillus [5]. While the secondary form can be caused by the retrograde spread from infected axillary lymph nodes, or by direct spread from tissue in the ribs, sternum, shoulder joint, costochondral cartilage, or pleura adjacent to the mammary gland tissue, the primary form is caused by the spread of infection through abrasions in the skin of the breast or through cracks in the nipple [15–22].

The incidence of breast tuberculosis is increasing due to the rise of underlying immunosuppressive diseases such as acquired immunodeficiency syndrome (AIDS) and the development of drug-resistant strains of *M. tuberculosis* [15, 23].

### Clinical features

Breast tuberculosis is a younger woman’s disease, occurring mainly in lactating and multipara women. It is much rarer in men, with a male/female ratio of 1/30 [24]. The patients’ general condition is good, and systemic findings such as fever, weight loss, night sweats, and anorexia are not generally observed [25].

**Fig. 1:**
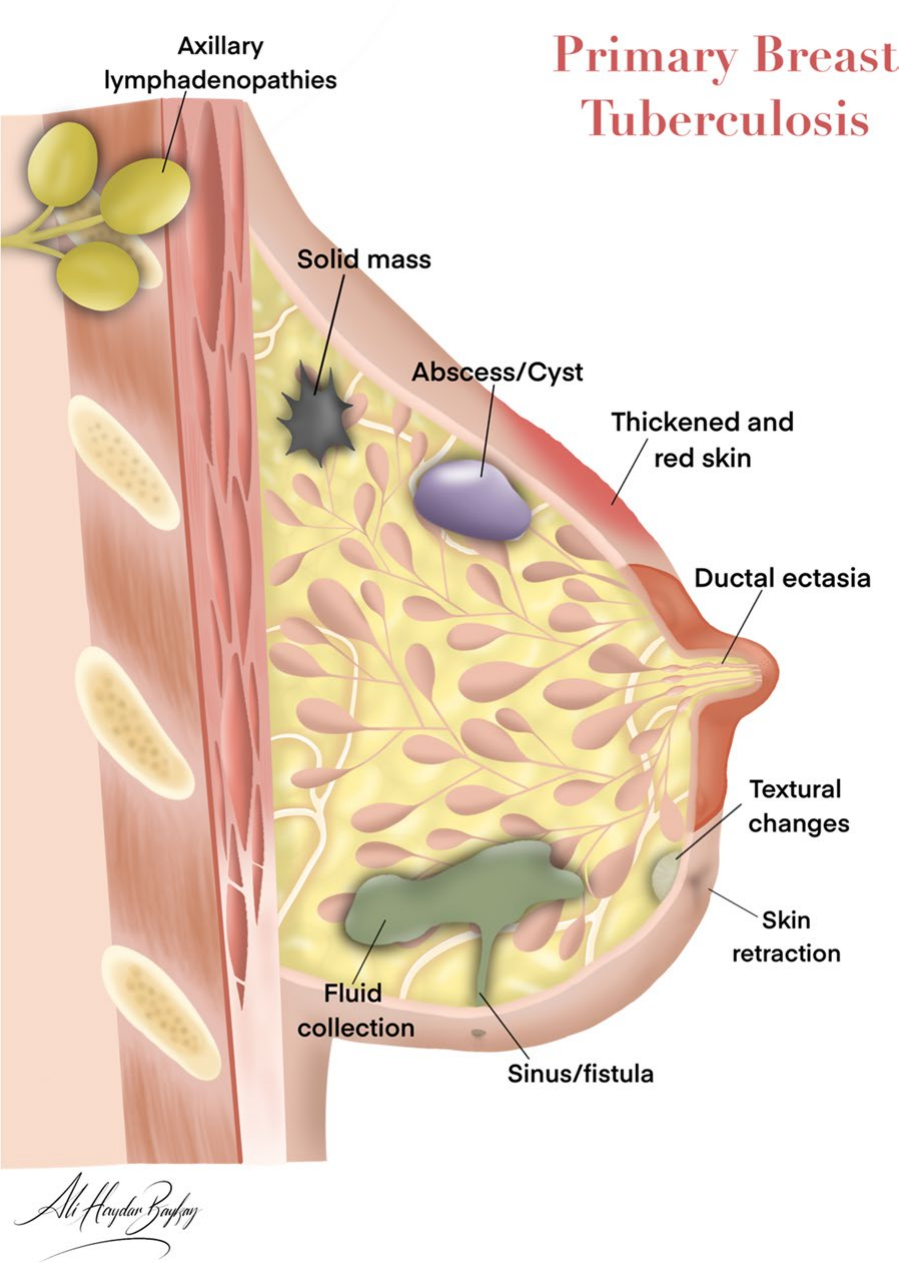
Breast anatomy and the effects of tuberculosis

**Fig. 2:**
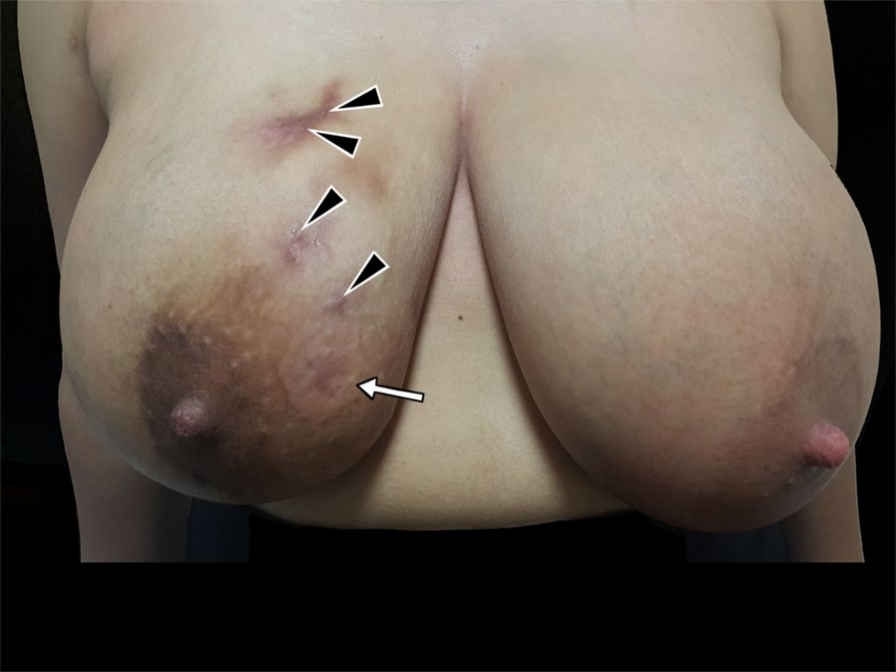
A 26-year-old female patient. Fistula orifices in the right breast (arrowheads) and erythema of the breast skin (arrow)

**Fig. 3:**
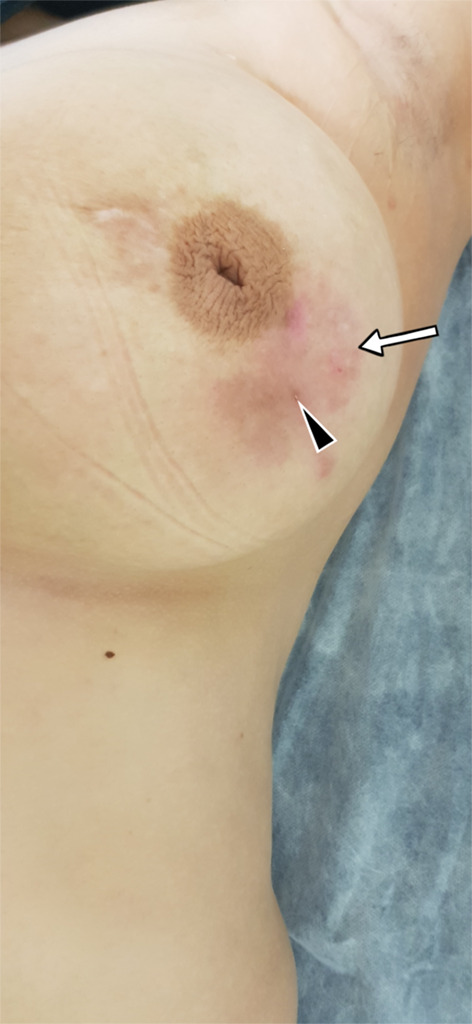
A 33-year-old female patient. Erythema of the skin in the left breast (arrow) and fistula orifice (arrowhead)

The clinical presentation is variable. However, in most cases, a painless palpable lump, which appears to be irregularly bounded, hard, and fixed to the skin or chest wall is often found in the upper outer quadrant or central area of the breast. Multiple lesions are uncommon, and the clinical presentation can simulate carcinoma [13, 15]. As a result, the findings are often indistinguishable from a carcinoma [15, 25]. Bilateral involvement has been reported in 3–30% of all cases [15, 25, 26]. Nipple and skin retractions, swellings, inflammatory changes, sinus formations, and axillary lymphadenopathies can also be observed (see Figs. 1, 2, 3), but breast discharge is rarely seen [12, 13, 15, 19, 26, 27].

Axillary lymph node involvement can be seen in 50–75% of cases [15, 20, 25] and the initial finding may be a pyogenic abscess [12, 13, 15, 19].

The findings are non-specific, so it is difficult to clinically distinguish between granulomatous mastitis and breast cancer [5, 9, 15]. The simultaneous coexistence of carcinoma and breast tuberculosis is rare and can lead to many problems regarding diagnosis and treatment [15].

### Classification

In 1952, McKeown and Wilkinson classified breast tuberculosis into five different types: nodular tuberculous mastitis, tuberculous mastitis obliterans, sclerosing tuberculous mastitis, disseminated tuberculous mastitis, and acute miliary tuberculous mastitis [4–6].

Nodular caseous tuberculous mastitis is the most common type. It presents a well-defined, slowly growing, painless, oval, non-vascular hypoechoic mass, mimicking the appearance of fibroadenoma in the early stages. Later, it may develop a fistula in the nipple-areolar complex or on the skin and more closely resemble cancer [4–6].

In tuberculous mastitis obliterans, the infection spreads along the mammary ducts, with epithelial proliferation, necrosis, and fibrosis eventually developing in the ducts. Occlusion-related hypoechoic and anechoic collections and debris in the ducts can have a ‘cystic mastitis’-like appearance. The appearances are similar to classic bacterial mastitis [4, 5, 9, 15].

Sclerosing mastitis is more prevalent among older women. Due to intense fibrosis and ill-defined textural change, nipple retraction and shriveling can be observed, and atrophy can eventually occur. It can mimic an inflammatory carcinoma [4–6, 9, 15].

Disseminated mastitis can often affect a large area or even the entire breast. Diffuse skin thickening, small fluid collections, multiple foci, and sinuses are also observed [4, 5, 9].

Acute miliary tuberculous mastitis is extremely rare and is hardly ever observed. It refers to the breast involvement of miliary tuberculosis disease [4, 5, 9].

In addition to this classification, Tewari and Shukla created an easy-to-use classification that is more suitable for current and daily practice [5]. According to this classification, breast tuberculosis appears in three forms: nodulo-caseous tubercular mastitis, disseminated tubercular mastitis, and tubercular breast abscesses [5].

### Imaging

Breast tuberculosis has three essential radiological appearances: nodular, diffuse, and sclerosing [23].

#### Chest X-ray

A chest x-ray may show signs of active or healed lung tuberculosis (in cases with secondary breast tuberculosis) as well as clustered calcified lymph nodes in the axilla caused by breast tuberculosis [15].

#### Mammography

The most common mammographic findings are diffuse trabecular thickening and skin retraction. An ill-defined breast mass can also be seen [15]. Nearly half (43.5%) of breast tuberculosis cases are reported as BIRADS 4/5 lesions [27].

The image size of the tubercular lesion shown on a mammogram is usually similar to its clinical size [5]. A nodular form of tuberculosis can resemble a fibroadenoma [5, 15].

**Fig. 4:**
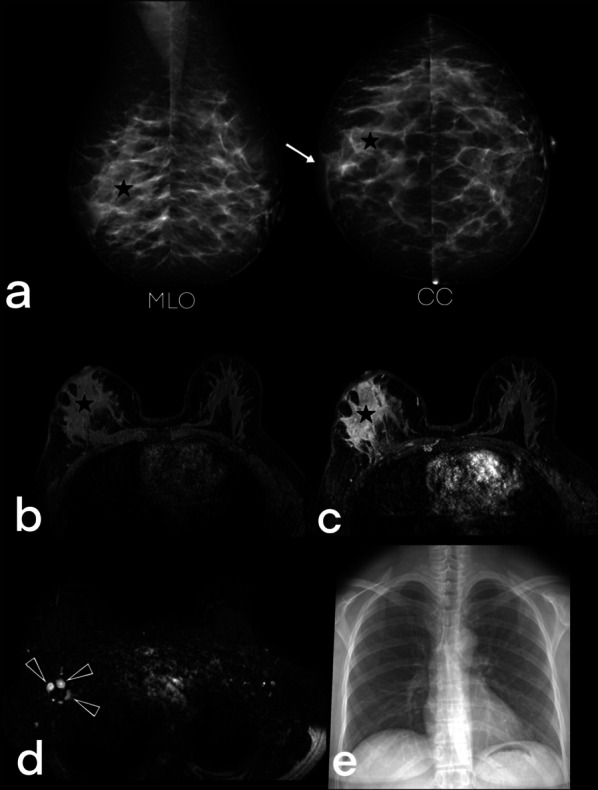
Mammograms of a 44-year-old female demonstrate disseminated tuberculosis in right breast (**a**) show an asymmetric opacity (star) and thickening of the skin (arrow). MRI images of the same patient: pre-contrast fat-suppressed T1-weighted (**b**), post-contrast fat-suppressedT1-weighted (**c**), and fat-suppressed T2-weighted images (**d**) show homogeneous non-mass enhancement with regional type distribution (star) and axillary lymph nodes (arrowheads). The chest x-ray (**e**) of the patient is negative for pulmonary tuberculosis. BIRADS 4

The diffuse form has the appearance of dense breast tissue with a diffuse increase in thickness (Fig. 4) on inflammatory carcinoma-like skin [5, 15]. This appearance is due to the fact that the infection is more virulent and/or the immune response is low [23].

The rare sclerosing form occurs in older women [27]. Due to extensive fibrosis, it is seen as a high-density breast mass accompanied by nipple retraction [5, 15]. Asymmetry between both breasts or retraction and reduction of breast volume in the affected breast is also observed. Finally, atrophy of the affected breast can occur [23], which typically reflects delayed diagnosis or poorly treated disease.

If there is localized skin thickness and sinus formation associated with an ill-defined breast mass, breast tuberculosis needs to be included in the differential diagnosis [15, 18]. Typically, benign calcifications (round or coarse) may also be seen by mammography, but suspicious calcifications are not expected.

#### Ultrasonography

Since breast tuberculosis is more common in young women aged 20–40, ultrasonography is often the first radiological examination method used in diagnosis. It is also the best method for evaluating axillary lymph nodes [5, 23]. Guided ultrasound is also very useful when performing a fine-needle aspiration (FNA), core needle biopsy, or percutaneous abscess drainage [5, 11, 15, 23].

**Fig. 5:**
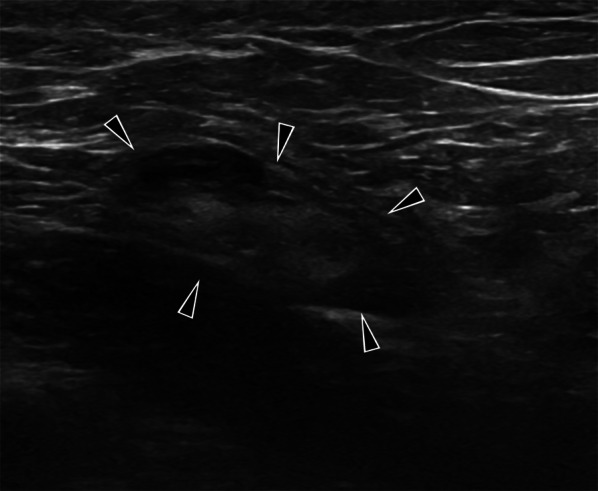
Ultrasound image of a 34-year-old female patient (she had a recently developed clinically suspicious palpable lesion) diagnosed with breast tuberculosis demonstrate nodular tuberculosis (arrowheads). The mass is observed as a well-circumscribed, oval-shaped, heterogeneous solid lesion. BIRADS 4

In the nodular form of the disease (Fig. 5), the lesion takes the form of an indistinct bordered, hypoechoic, heterogeneous mass [5, 15]. If the patient’s immunity is high and the disease’s virulence is low, a slow-growing, well- circumscribed, hypoechoic, posteriorly enhanced solid lesion, similar in appearance to a slowly growing fibroadenoma can be observed [23] and categorized as BIRADS 3 lesion.

**Fig. 6:**
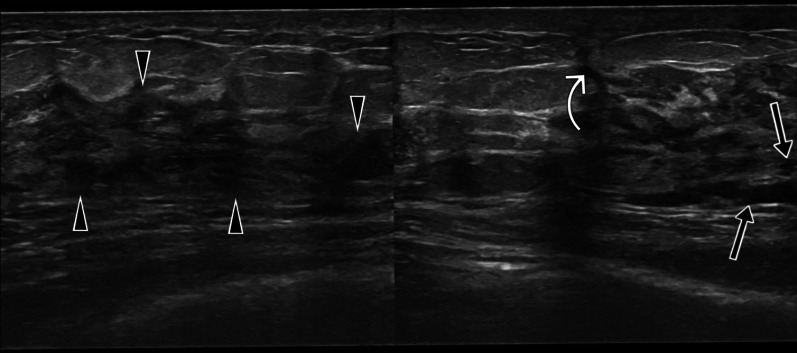
Ultrasound images of a 33-year-old female. Parenchymal diffuse hypoechoic coarsening in the left breast (arrowheads), small fluid collections (arrows), and sinus tract (curved arrow). BIRADS 4

**Fig. 7:**
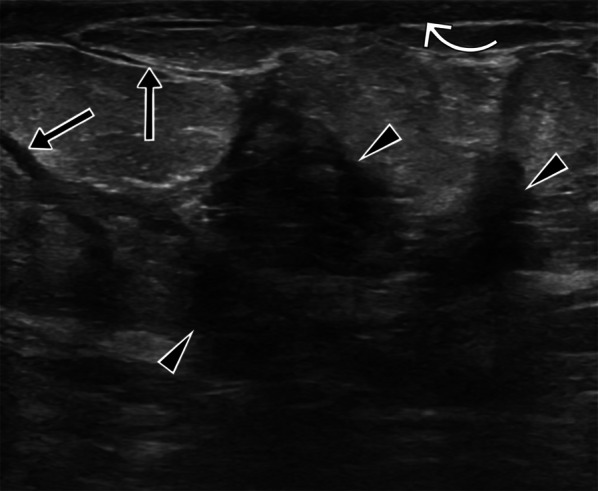
A 24-year-old and 30-weeks pregnant female patient. Ultrasound images show parenchymal edema in the right breast and abscesses with dense content (arrowheads), fistula tracts (arrows), and thickening of the skin (curved arrow). BIRADS 4

While ill-defined hypoechoic masses are observed in the diffuse form (Figs. 6, 7) an increase in the echogenicity of the breast parenchyma without a mass formation is typical for the sclerosing form [7, 19]. This echogenicity increase reflects edema and inflammation.

Increases in focal (skin bulge) and diffuse (due to edema) thickness, as well as ductal ectasia, can also be detected by ultrasound [5, 12, 15, 23].

**Fig. 8:**
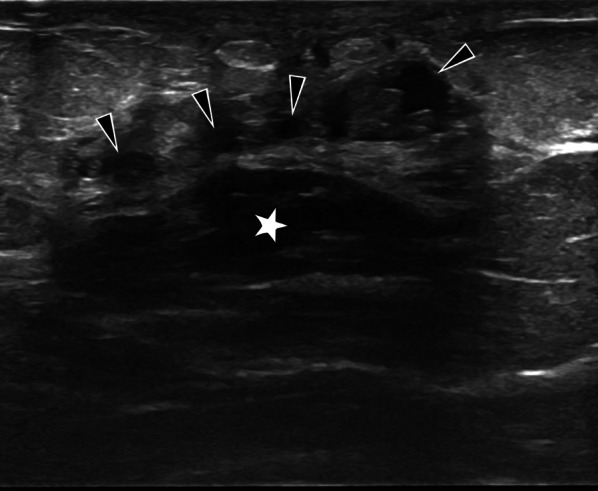
A 46-year-old female with PCR (polymerase chain reaction) proven tuberculosis in the left breast. Gray-scale ultrasound image shows multiple abscesses (arrowheads) and a hypoechoic, oval mass with circumscribed margins (star). BIRADS 4

**Fig. 9:**
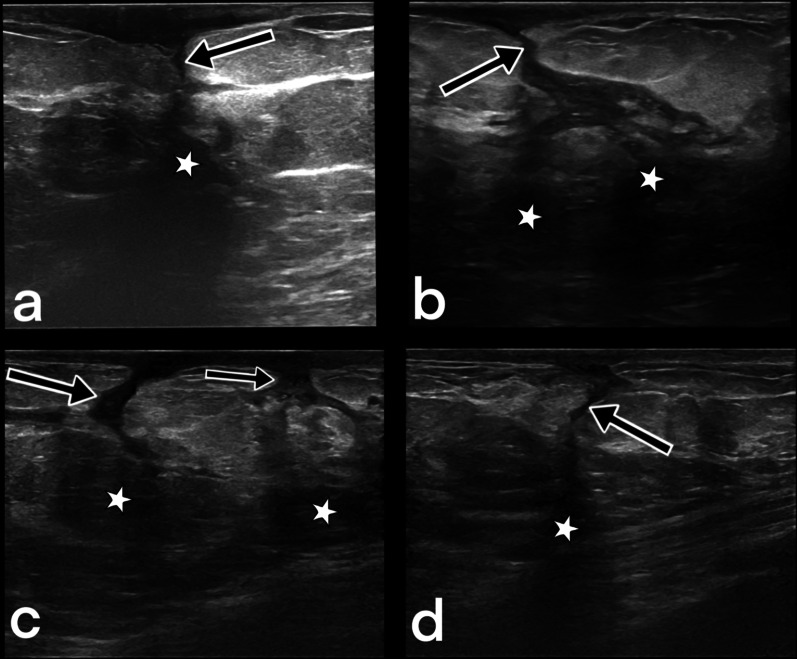
Ultrasound images from four different female patients (**a**–**d**). Sinus tracts (arrows) and related abscess formations (stars). BIRADS 4

Ipsilateral axillary adenopathy is present in 20–69% of cases [9, 23, 27]. The lymph nodes have a round or oval shape, are smooth-edged, and enlarged (short axis > 1 cm or the cortical thickness > 5 mm) [9, 23]. The presence of a fatty, echogenic hilus and an oval shape are useful features in distinguishing tuberculosis lymphadenitis from malignancy [23]. Conglomerate lymph node masses or fistulas may also be seen [5, 15], as well as a large hypo-anechoic, fluctuating abscess with dense content and internal echogenicity [15, 23]. Septations are uncommon, but multiple small abscesses interrelated with each other may also develop (Fig. 8). If an abscess fistulizes to the skin, the fistula tract (Fig. 9) may also be visible with ultrasound [5, 9, 11, 12, 15, 23].

#### Computed tomography (CT)

CT is useful for evaluating lesions with a deep retromammary localization and with thoracic wall involvement.

Abscesses appear on CT as well-defined lesions showing rim-like peripheral contrast enhancement. In addition, CT may indicate a fistula opening to the pleura, or infected ribs, bone structures, or lung parenchyma. Percutaneous drainage of a deep-seated abscess is possible under CT guidance [5, 9, 12, 15].

#### Magnetic resonance imaging (MRI)

**Fig. 10:**
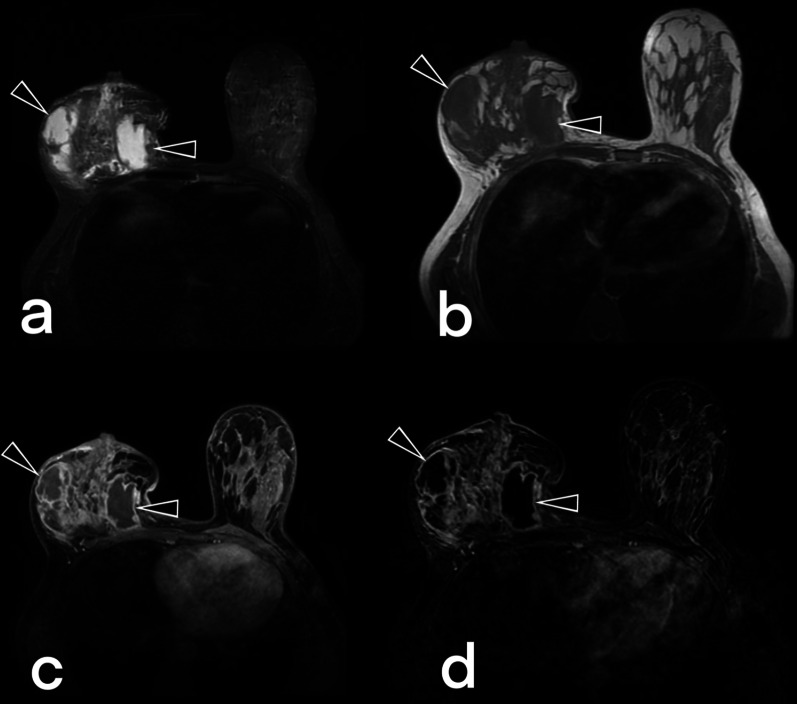
Breast MRI images of a 39-year-old female. T2 weighted fat-suppressed image (**a**), T1 weighted image (**b**) post-contrast (**c**), and subtracted image (**d**) obtained at the late phase after intravenous gadolinium injection demonstrate multiple fluid collections (arrowheads) with peripheral enhancement in the right breast in keening with abscess. Diffuse involvement. BIRADS 4

**Fig. 11:**
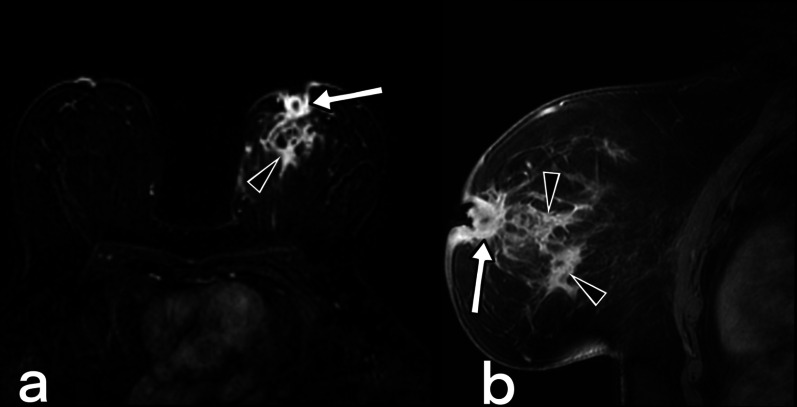
MRI images of a 37-year-old female. Axial (**a**) and sagittal (**b**) fat-suppressed T1 weighted contrast-enhanced images demonstrate nodular tuberculosis in the left breast. MR image shows an irregular shaped spiculated margins mass (arrowheads) with heterogeneous internal enhancement. The lesion mimics a breast carcinoma with nipple retraction (arrows). BIRADS 5

**Fig. 12:**
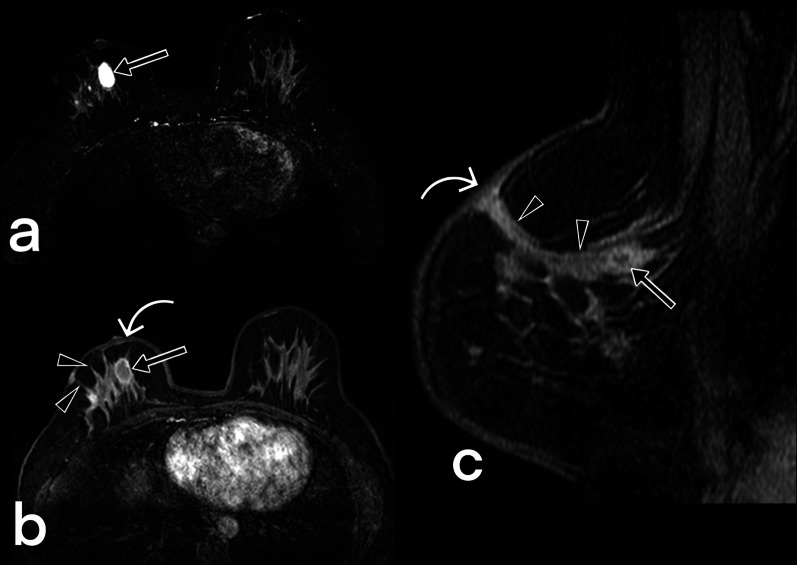
MRI images of a 41-year-old female. Axial T2 weighted fat suppressed (**a**), fat-suppressed T1 weighted contrast-enhanced axial (**b**), and sagittal (**c**) images reveal cystic lesions (arrows) in the right breast with peripheral enhancement, compatible with an abscess that is fistulized (arrowheads) to the thickened skin (curved arrow). BIRADS 4

**Fig. 13:**
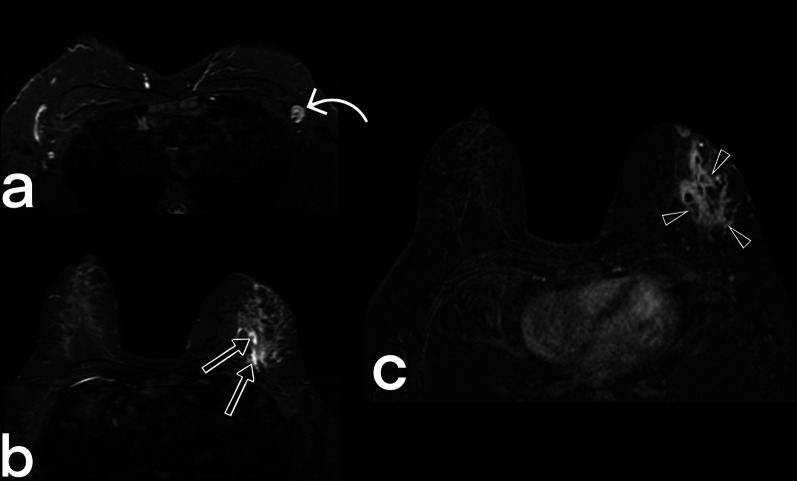
MR images of a 32-year-old female. Axial T2-weighted fat-suppressed (**a**, **b**) and T1-weighted fat-suppressed contrast-enhanced images (**c**) reveal a signal increase and heterogeneous non-mass enhancement with regional type distribution in the left breast (arrowheads), and abnormal axillary lymph nodes (curved arrow). Small fluid collections are also noted (arrows). BIRADS 4

Breast tuberculosis lesions are typically hyperintense on T2-weighted images. Post-contrast T1-weighted MR images show nonspecific enhancement of the breast parenchyma and rim-shaped enhancement of the abscess wall. Sinus formations that are not depicted on ultrasonography and mammography can be demonstrated with MRI. MRI can show the extension of an abscess into extramammary areas [5, 11, 12, 15, 23]. MRI is also a useful modality for showing the continuation of the fistula tract into deep tissues (Figs. 10, 11, 12, 13).

### Biopsy

Three methods are used to take samples: FNA (the most common primary invasive diagnostic method), core needle biopsy, and open biopsy [15].

The pathological diagnosis of breast tuberculosis is not always easy. Necrotizing granulomatous inflammation in FNA samples can be detected in approximately 73% of cases [5]. The prevalence of acid-fast bacilli (AFB) ranges from 0 to 38.6% [3]. Despite this low prevalence, growing tuberculosis bacilli in the Ziehl–Neelsen stain is still considered the gold standard for diagnosis [3].

A core needle biopsy provides a better sample and is sometimes necessary to confirm the diagnosis and exclude breast cancer [15]. An open biopsy from the mass, an abscess wall, a sinus, or an ulcer area can almost always confirm breast tuberculosis [5, 15]. If clinical and radiological findings support breast tuberculosis, a core needle biopsy is usually sufficient for a positive diagnosis [5, 27]. An open biopsy is rarely needed.

**Fig. 14:**
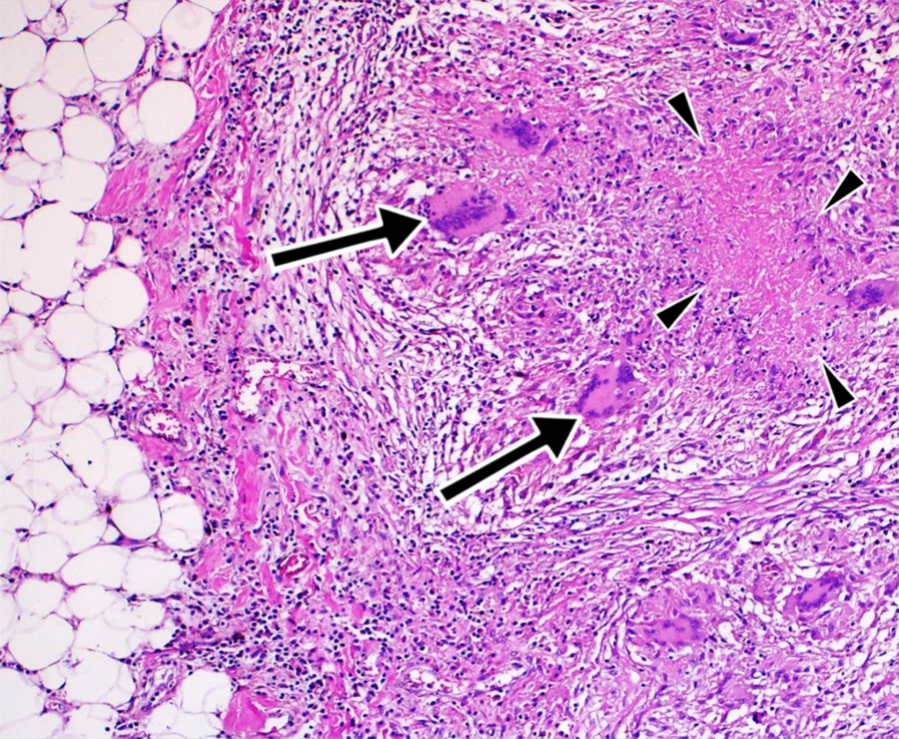
Histopathologic evaluation (Hematoxylin Eosin × 20) of a tru-cut biopsy specimen demonstrates granuloma with Langshan’s giant cells (arrows) and caseous necrosis (arrowheads) in lipomatous tissue, compatible with tuberculosis

Histologically, breast tuberculosis is a form of granulomatous inflammation (Fig. 14). Sarcoidosis, granulomatous mastitis, fungal infections such as actinomycosis, plasma cell mastitis, and traumatic fat necrosis may show a tuberculoid-type tissue reaction indistinguishable from tuberculosis [5, 15]. Tuberculous mastitis can be diagnosed reliably by cytological evidence of epithelioid cell granulomas, lymphohistiocytic aggregates and Langhans’ giant cells with or without caseous necrosis [25, 26].

Idiopathic granulomatous mastitis is an inflammatory reaction consisting of epithelioid multinuclear giant cells, leukocyte infiltration and abscess, but caseous necrosis is not observed. Granulomas are restricted by breast lobules. The distribution of granulomas in breast tuberculosis is common and not limited to the lobules. Histologically traumatic fat necrosis is confined to the broken down fat globules. In plasma cell mastitis, plasma cells and giant cells accumulate in dilated ducts. The presence of sulfur granules at sites of infection is a typical histopathological change for actinomycosis [25].

### Treatment and follow-up

**Fig. 15:**
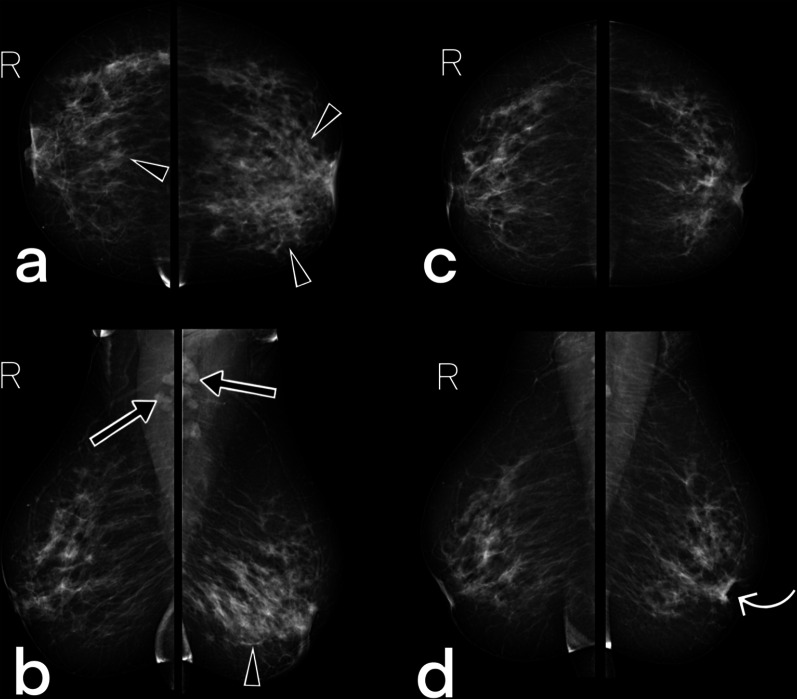
A 42-year-old female (she had a recently developed palpable lesion) with PCR (polymerase chain reaction) proven tuberculosis in both breasts. Pre-treatment CC (**a**) and MLO (**b**) mammograms demonstrate a focal (right breast), global (left breast) asymmetry (arrowheads), and diffuse trabecular thickening (BIRADS 4). Note bilateral axillary lymph nodes (arrows). Ultrasound images show parenchymal focal edema (US images are not included in the figure). On the mammograms obtained after antituberculous therapy of 12 months, bilateral breast parenchyma appear normal, and lymph nodes are not visible. Note the retraction of the left nipple (curved arrow). BIRADS 2

**Fig. 16:**
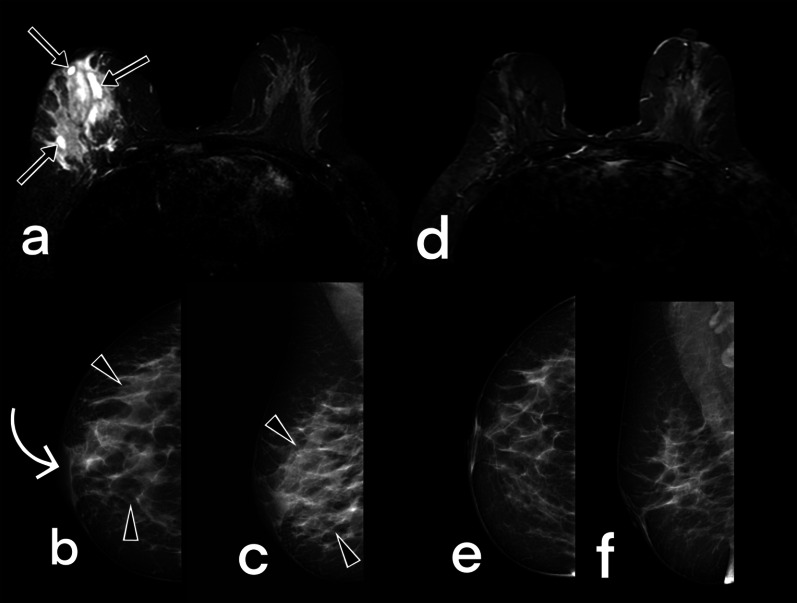
Images of a 44 years-old female patient obtained before (**a**–**c**) and 12 months after (**d**–**f**) antituberculous therapy. T2-weighted fat-suppressed axial image (**a**) demonstrates fluid collections (arrows) and a diffuse signal increase in the right breast. Mammograms (**b**, **c**) show a diffuse increase (BIRADS 4) in parenchymal opacity (arrowheads) and thickening of the skin (curved arrow). After the treatment, complete regression (BIRADS 1) of the findings is observed (**d**–**f**)

There is no medical treatment guide specific to breast tuberculosis. Like pulmonary or extrapulmonary tuberculosis treatment, breast tuberculosis is treated by 6–18 months of anti-tubercular chemotherapy (Figs. 15, 16). Surgery (lumpectomy or mastectomy) is also an option when there is no response to medical treatment [2, 3, 5, 25]. Abscesses can be effectively treated with ultrasound-guided external drainage. Patients generally respond well to medical therapy and do not need a second biopsy during follow-up.

## Conclusion

Breast tuberculosis is a difficult disease to diagnose, and clinical evaluation, radiological examination, and histopathological and microbiological sampling are often necessary to confirm diagnosis. Radiological examinations are not only important in diagnosing the disease but are also important during the treatment and in the follow-up.

## Data Availability

Not applicable.

## Abbreviations

AFB: Acid-fast bacilli
BIRADS: Breast Imaging-Reporting and Data System
CT: Computed tomography
FNA: Fine-needle aspiration
MRI: Magnetic resonance imaging
